# Giant reversible anisotropy changes at room temperature in a (La,Sr)MnO_3_/Pb(Mg,Nb,Ti)O_3_ magneto-electric heterostructure

**DOI:** 10.1038/srep27501

**Published:** 2016-06-08

**Authors:** Rajesh Vilas Chopdekar, Michele Buzzi, Catherine Jenkins, Elke Arenholz, Frithjof Nolting, Yayoi Takamura

**Affiliations:** 1 Department of Materials Science and Engineering, Univ. of California, Davis, Davis, CA 95616, USA; 2 Swiss Light Source, Paul Scherrer Institute, CH-5232 Villigen PSI, Switzerland; 3 Advanced Light Source, Lawrence Berkeley National Laboratory, Berkeley, CA 94720, USA

## Abstract

In a model artificial multiferroic system consisting of a (011)-oriented ferroelectric Pb(Mg,Nb,Ti)O_3_ substrate intimately coupled to an epitaxial ferromagnetic (La,Sr)MnO_3_ film, electric field pulse sequences of less than 6 kV/cm induce large, reversible, and bistable remanent strains. The magnetic anisotropy symmetry reversibly switches from a highly anisotropic two-fold state to a more isotropic one, with concomitant changes in resistivity. Anisotropy changes at the scale of a single ferromagnetic domain were measured using X-ray microscopy, with electric-field dependent magnetic domain reversal showing that the energy barrier for magnetization reversal is drastically lowered. Free energy calculations confirm this barrier lowering by up to 70% due to the anisotropic strain changes generated by the substrate. Thus, we demonstrate that an electric field pulse can be used to ‘set’ and ‘reset’ the magnetic anisotropy orientation and resistive state in the film, as well as to lower the magnetization reversal barrier, showing a promising route towards electric-field manipulation of multifunctional nanostructures at room temperature.

While single phase multiferroic materials exist in nature, often they have relatively weak magnetoelectric coupling and their ordering temperatures are well below ambient conditions. In an effort to develop room-temperature magnetoelectric functionality, there has been a strong thrust of research in the direction of artificial multiferroics consisting of composite structures^1^,^2^. A magnetoelectric property from cross coupling of different ferroic phases may arise due to a structural interaction as in magnetostrictive-piezoelectric composites^3^, or may be produced from an electric charge-based phenomenon such as through the use of a gate voltage in proximity to a ferromagnetic channel^4^. This emergent magnetoelectric functionality has been recently implemented in so-called ‘straintronic’ device designs to minimize active power consumption as compared to more conventional current-assisted nanomagnet memory writing schemes^5^,^6^.

Imaging of the local control of magnetic properties via strain has been recently demonstrated in magnetic materials such as Fe^7^, CoFe^8^, spinel ferrites^9^, or FeRh^10^ coupled to ferroelectric (FE) substrates such as BaTiO_3_ (BTO). Similarly, epitaxial ferromagnetic manganite films have been used as prototypical materials showing coincident dynamic tuning of multiple functional properties when coupled to BTO^11^,^12^,^13^. Spatially resolved studies of the manganite-BTO system illustrate that by changing the magnitude and symmetry of strain by traversing through BTO phase transitions, a persistent 45° or 90° rotation of magnetization occurs at the scale of individual magnetic domains^14^. Additionally, devices utilizing doped manganites have shown current-driven switching of magnetization as well as nearly full spin polarization^15^,^16^. This strong link between structure, ferromagnetism, and spin polarization can be an avenue for electric field control of spin transport in manganite-based nanoscale heterostructures.

Recent work has shown that a large, anisotropic, and hysteretic in-plane strain may be generated from the (011) crystal orientation of the relaxor FE [Pb(Mg_1/3_Nb_2/3_)O_3_]_(1−x)_-[PbTiO_3_]_x_ (PMN-PT) with x = 0.32 near the morphotropic phase boundary^17^. Using these substrates, changes in magnetization of polycrystalline metallic films have been induced with an applied electric field and can be correlated to the anisotropic in-plane strain generated from FE domain reconfiguration^18^. This phenomenon can be used to re-orient the magnetization of sub-micron sized Ni nanostructures with strong shape-induced magnetic anisotropy^19^. Furthermore, the use of an 80–100 nm thick single crystalline manganite layer in lieu of a polycrystalline film leads to a strain-based magnetoelectric effect through the rotation of in-plane magnetic anisotropy by up to 22 degrees, as well as hysteretic resistivity tunable though the strain state of the film^20^,^21^. An analysis of the reversible electric-field switching behavior of the manganite/PMN-PT system through X-ray spectroscopy techniques as well as theoretical calculations show that the induced strain change leads to a change in Mn e_g_ orbital population preference, causing a reversible shift in Curie temperature of up to 10 K^22^.

We show here that a model epitaxial perovskite system consisting of a 17 nm thick (011)-oriented La_0.7_Sr_0.3_MnO_3_ (LSMO) film on a PMN-PT substrate (Fig. 1(a)) exhibits robust room temperature strain-mediated magnetoelectric coupling manifesting as non-volatile changes in magnetic domain structure, magnetic anisotropy, and resistivity. To probe such changes, we use chemically and spatially resolved techniques such as X-ray magnetic circular dichroism (XMCD) spectroscopy and X-ray photoemission electron microscopy (PEEM) under constant applied electric fields as well as electric and magnetic field pulses. We directly correlate FE domain re-configuration and thus dynamically tuned film strain state with large and abrupt changes in the magnetic domain structure and film resistivity.

## Results

### Film-averaged structural and functional characterization

Following the notation of Wu *et al.*^18^, we distinguish between the eight possible variants of PMN-PT domain orientation in the following manner: the four 〈111〉 FE orientations that lie wholly in the (011) plane belong to the P_xy_ poling state, and the four 〈111〉 orientations that lie partially out-of-plane are either termed as P_z+_ or P_z−_ poling state depending on the direction of the out-of-plane FE component. An electric field pulse of approximately 2 kV/cm is sufficient to rotate the FE polarization from P_xy_ to P_z_ or vice versa. Due to the small Thompson-Fermi screening length of order one unit cell compared to the film thickness^23^, little change in film behavior is observed due to charge accumulation or depletion at the film/substrate interface, with a saturation magnetization change of 2% in a 17 nm thick LSMO film at 10 K with the PMN-PT poled in the P_z−_ state compared to the P_z+_ state. Thus, we concentrate on studying the effects of transitions from the P_xy_ to P_z_ states and vice versa at room temperature.

An analysis of the change in LSMO unit cell dimensions upon a change in FE domain configuration was performed by poling the substrate into a series of different configurations and measuring the change in position of seven out-of-plane and partially in-plane reflections with X-ray diffraction-based reciprocal space mapping. The film peak positions for the P_xy_ configuration were best fit to a monoclinic unit cell, with the average lattice parameter **a** along the [100] substrate direction as 0.3894 ± 0.0002 nm and partially out-of-plane parameters **b** and **c** as both 0.3890 nm within the error of the measurement. The monoclinic angle between **b** and **c** shows a small change from 90.4° ± 0.1° in the P_xy_ state to 90.2° in the P_z−_ state. The change in PMN-PT and LSMO unit cell dimensions along orthogonal in-plane and out-of-plane directions as a function of substrate poling state is compared to a macroscopic strain gauge measurement in Supplementary Table S1.

While the 17 nm thick LSMO film has a significant static distortion compared to the bulk pseudocubic parameter the film is partially relaxed due to the large mismatch with the PMN-PT substrate (pseudocubic lattice parameter of approximately 0.387 and 0.402 nm for LSMO and PMN-PT, respectively). However, we observe a large reversible change in LSMO lattice parameter compared to its bulk values when the substrate FE state is changed from P_xy_ to P_z−_, with an anisotropic strain change of 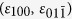 = (0.41%, 0.67%) for the P_xy_ state and (0.43%, 0.44%) for the P_z−_ state. The LSMO film is in tension along both the [100] and [01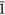] crystallographic directions, but the change in magnitude of tension is most significant along the [01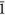] direction when changing between the P_xy_ and P_z_ states (see Supplementary Figure S1 and Table S2). A static anisotropic distortion is seen in films grown on dielectric substrates due to the anisotropic elastic moduli of bulk (La,Sr)MnO_3_^24^, but the large change in dilational strain along the [01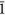] direction allows for tuning of the film strain from a highly anisotropic to a nearly isotropic strain state.

In contrast to previous studies of almost fully-relaxed LSMO films on PMN-PT (e.g. film thickness of 80–100 nm)^20^,^21^, our system has a static distortion of the LSMO unit cell in the as-grown state in addition to the imprinted strain generated upon reorientation of the substrate FE domains. To disentangle the effects of epitaxial mismatch from electrostrictive or ferroelastic domain contributions due to the applied electric field, we first examine macroscopically averaged functional properties such as sheet resistivity and magnetization and compare these to previously reported studies. Due to the double exchange mechanism responsible for the ferromagnetic order in manganites, there is a coincident metal-insulator transition (MIT) accompanying the paramagnetic-ferromagnetic transition at the Curie temperature (T_c_)^25^. Both finite size effects as well as epitaxial strain as derived in the Millis model^26^ may lower the Curie temperature of LSMO as compared to the bulk value of approximately 370 K. For the 17 nm LSMO film, the metal-insulator transition (T_MIT_) for the as-grown state in zero magnetic field is 322 ± 1.3 K and the T_c_ as measured by superconducting quantum interference device (SQUID) magnetometry is 322 ± 1.6 K. Furthermore, there is a lowering of T_c_ by approximately 3 K when the sample is poled from the P_z_ state to the P_xy_ state. The suppression of T_c_ and T_MIT_ due to the static epitaxial strain or finite size contributions can be expected due to the large lattice mismatch between LSMO and PMN-PT. However, we can clearly resolve the influence of substrate FE domain configuration on the ferromagnetic and resistive properties through sequential poling of the substrate at room temperature.

**Figure 1:**
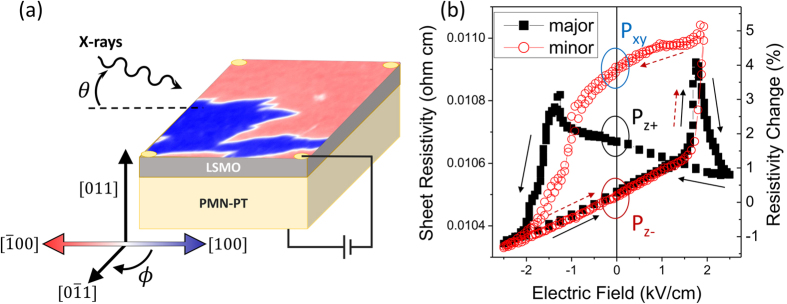
(**a**) Schematic and experimental geometry of the artificial multiferroic sample, with color scale indicating the in-plane magnetization orientation in the P_xy_ poled state as obtained from PEEM. (**b**) Electric-field dependence of the LSMO film sheet resistivity at 298 K for a major bipolar loop sweeping from the P_z−_ state to the P_z+_ state as well as a minor loop poling from the P_z−_ to the P_xy_ state over two cycles, showing clear hysteresis in resistivity at zero applied electric field. Solid arrows indicate sweep direction for the major loop, and dashed arrows indicate sweep direction for the minor loop.

In Fig. 1(b), the resistivity of the LSMO film in the metallic state at 298 K is shown as a function of applied electric field. Both a major hysteresis loop (±2.5 kV/cm, showing transitions from P_z−_ to P_xy_ to P_z+_ states) and a minor loop (−2.5 kV/cm to 1.8 kV/cm, showing transitions only between P_z−_ to P_xy_ states) are plotted. Peaks in the major loop correspond to FE axis rotations from P_z_ to P_xy_ or vice versa, causing an increase in in-plane strain and thus an increase in Mn-O bond distance as exhibited by the increase in 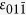 magnitude measured from reciprocal space mapping. Due to this change, the Mn e_g_ hopping integral decreases and the resistivity increases. Comparison between the major and minor loops illustrates that the bistable strain at zero applied electric field produces large non-volatile room temperature changes in resistivity (3.8% change from P_z−_ to P_xy_ in Fig. 1(b)). The magnitude of the resistivity peaks is an increase over previously reported values of approximately 1%, likely due to the larger electric-field induced changes in unit cell dimensions compared to thick relaxed films^21^. The sensitivity of the LSMO resistivity to strain is also illustrated by the measurable slope between −2.5 kV/cm and 0 kV/cm where no significant FE axis reorientation takes place but there is a small change in FE axis angle due to the converse piezoelectric effect.

**Figure 2:**
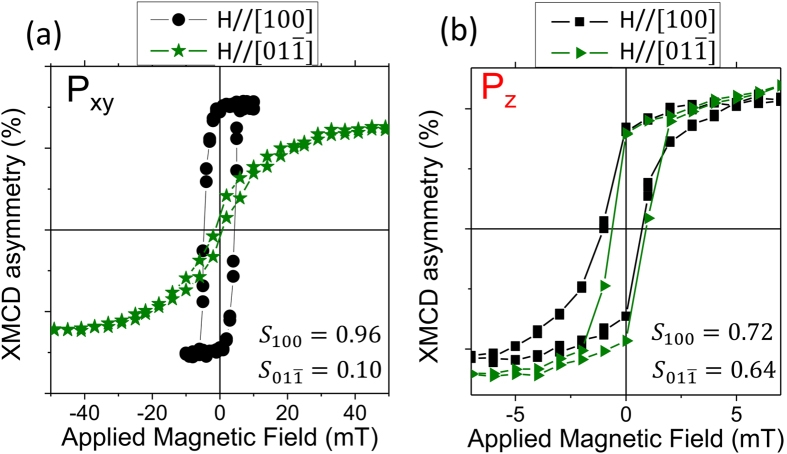
Mn *L*_*3*_ XMCD hysteresis loops taken at 298 K measured in zero electric field with the substrate poled in the (**a**) P_xy_ and (**b**) P_z+_ states. The hysteresis loops average over a sample area of order 100 microns in diameter, and are acquired with the magnetic field along two orthogonal in-plane directions, [100] and [01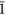]. The [100] direction has high remanence and loop squareness S = M_rem_/M_sat_ when poled in the P_xy_ state, but the sample is nearly isotropic when poled in either the P_z+_ or P_z−_ state.

Macroscopically averaged XMCD hysteresis loops taken at the Mn *L*_*3*_ edge (Fig. 2) show that a large two-fold in-plane magnetic anisotropy exists for the P_xy_ state, with the easy axis corresponding to the [100] in-plane direction and a coercive field of 4 mT. Loop squareness as calculated from the ratio of the magnetization at remanence to the saturation magnetization (S = M_rem_/M_sat_) is high along the [100] direction, but is significantly reduced along the [01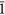] direction as shown in Fig. 2. This anisotropic behavior is similar to that of (011)-oriented LSMO films grown on SrTiO_3_ substrates, with a 250 nm thick film having a uniaxial magnetoelastic anisotropy constant of K = 8.4 × 10^4^ erg/cm^3 ^27. For the P_xy_ poling state (Fig. 2(a)), the hard [01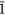] hysteresis loop can be used to estimate an effective anisotropy constant of K_eff_ = 3.3 × 10^4^ erg/cm^3^. However, upon rotation of the FE domains to the P_z_ state (Fig. 2(b)), the hysteresis behavior is more isotropic and the effective anisotropy constant is reduced to below 8 × 10^3^ erg/cm^3^. As the strain along the [100] direction is not changed significantly by reorientation of the PMN-PT domains (see Supplementary Table S1), we correlate this change in magnetic anisotropy to the reduction in strain along the [01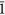] direction for the P_z_ FE domain state.

### Spatially-resolved measurements of magneto-electric coupling

**Figure 3:**
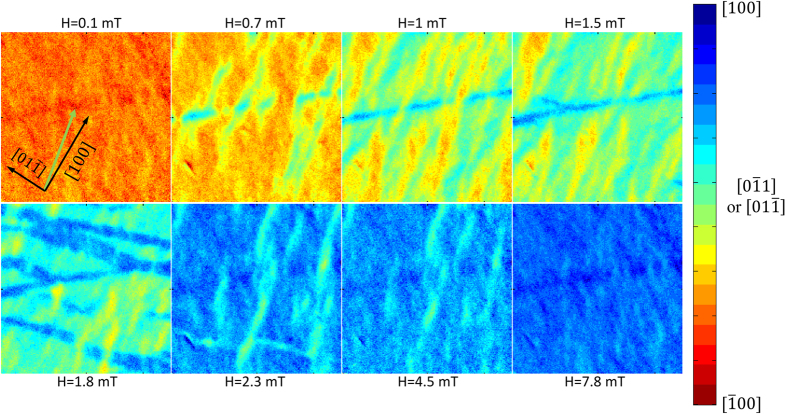
Montage of colorized PEEM asymmetry images at 298 K in a 15 × 15 μm region as a function of applied magnetic field pulses with the substrate poled in the P_z+_ configuration and initial applied magnetic field along the [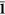
00] direction. The magnetic field pulse and X-ray incidence direction are collinear and are indicated by the green arrow in the upper left panel, with in-plane crystallographic directions shown for reference. Magnetization reversal occurs first by nucleation of many small domains oriented along the [01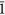] or [0
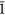
1] directions, followed by domain wall motion and subsequent rotation of magnetization towards the [100] direction.

We now compare spatially-averaged hysteresis loops with spatially-resolved PEEM measurements to locally map individual magnetic domains and their response to either electric or magnetic field pulses. Figure 3 is a series of PEEM images for a P_z+_ poled state with a color scale proportional to the magnetization direction derived from XMCD asymmetry measurements and is used to distinguish between magnetization strongly aligned along the X-ray propagation direction and nearly collinear with the [100] or [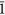
00] directions (blue and red, respectively), and magnetization oriented orthogonal to the X-ray propagation direction and aligned along the [01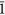] or [0
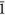
1] directions (light green). The initial magnetic field pulse orients the LSMO domains in the field of view along the [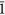
00] direction, but careful comparison of the contrast variation in the field of view of a single image, combined with vector magnetization measurements (Supplementary Figure S2) and comparison of the field of view for a P_xy_ poled state reveals that the domain magnetization in the P_z_ poled state cants away from the [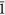
00] direction. This in-plane canting angle ranges between 10 and 40 degrees as seen by the orange and yellow domains in the top left panel of Fig. 3, whereas magnetization completely along the [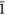
00] direction would have a dark red color. This canting of magnetization is seen macroscopically in Fig. 2 as the P_xy_ hysteresis loop has S_100_ = 0.96, but for the P_z_ poled state, the remanent magnetization is significantly lower than the saturation magnetization.

A small positive magnetic field of 0.7 mT nucleates many small domains whose magnetization is most closely oriented along the 〈01
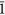
〉 directions. These initial nucleated domains are on the scale of 1–2 μm, similar in size to the average domain size of PMN-PT FE domains as measured by piezoelectric force microscopy^19^,^28^. Subsequent stronger field pulses cause domain wall motion along the applied field direction, but after a field pulse of 1.5 mT, a majority of the domains are oriented orthogonal to the applied field. Higher field values are required to rotate the magnetization of these domains to align with the [100] direction, and only after a field pulse of 7.8 mT does the field of view become well oriented towards the [100] direction. Note that this field value is similar in magnitude to the field required to bring the P_z_ poled sample to saturation in Fig. 2. Thus, magnetization reversal occurs through sequential non-180° magnetization rotations for the P_z_ poled configuration.

**Figure 4:**
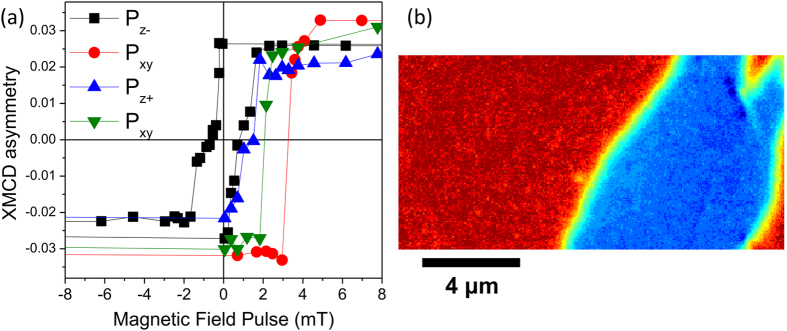
(**a**) Median XMCD asymmetry along the [100] direction for a 1 × 1 μm region as a function of applied magnetic field pulse at 298 K. Four different substrate poling states are shown, taken in a sequence of poling conditions from P_z−_, P_xy_, P_z+_, and returning to P_xy_. The area is taken from the center of the location shown in Fig. 3. (**b**) PEEM image after a magnetic field pulse of H = 3.8 mT showing reversal of magnetization in the field of view by nucleation of a single large domain for the P_xy_ poled state. This region is the same location as the upper half of the field of view shown in Fig. 3, utilizing the same color scale indicating magnetization direction.

A spatial average of the PEEM XMCD asymmetry measured in remanence is plotted in Fig. 4 as a comparison to the hysteresis loops presented in Fig. 2. The same 1 × 1 μm region was used for four magnetic hysteresis loops taken in sequence of poling conditions from P_z−_, P_xy_ (see Supplementary Figure S3 for corresponding images), P_z+_, and returning to P_xy_. The complete hysteresis loop is plotted for the P_z−_ state, and only the negative to positive magnetic field sweep is plotted for the subsequent states. LSMO magnetic domain reversal for the P_xy_ state (red circles and green diamonds in Fig. 4(a)) is markedly different than the series of images shown in Fig. 3, with reversal occurring by nucleation of a single 180° reversed area followed by domain wall motion. The stochastically determined field required to nucleate the reversed domain ranges between 3 and 4 mT from the PEEM measurements, which is close to the coercive field obtained from the XMCD hysteresis loop in Fig. 2.

**Figure 5:**
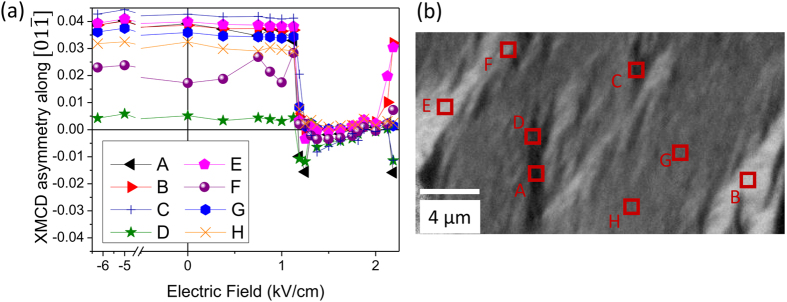
(**a**) Comparison of median XMCD asymmetry at 298 K along the [01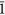] direction for eight 1 × 1 μm regions (indicated in the bottom panel) on poling the LSMO/PMN-PT sample from the P_z−_ state to the P_z+_ state. The PEEM images show clear suppression of contrast along the [01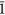] direction when the substrate is poled from the P_z−_ to the P_xy_ configuration. (**b**) PEEM image taken at a field of 2.2 kV/cm having a mixture of P_xy_ (regions A–F) and P_z+_ (regions G and H) in the field of view.

As the electric-field induced anisotropy shifts from nearly isotropic to strongly two-fold symmetry, we postulate that an electric field driven magnetization rotation of 90° or even 180° is possible. By saturating the LSMO sample in a positive magnetic field and sweeping an electric field from −6.5 kV/cm to + 2.5 kV/cm (Fig. 5(a)), the PMN-PT domain state starts in the P_z−_ state, then switches to P_xy_ and finally is on the verge of the P_z+_ state. Median XMCD asymmetry for 1 × 1 μm areas are plotted in Fig. 5, illustrating a sharp change in contrast at approximately 1.4 kV/cm and 2 kV/cm field values. The initial magnetization state, with a significant number of domains aligned towards the [01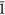] direction, is rotated by 90° to lie along the 〈100〉 directions at an electric field of 1.5 kV/cm, and upon further poling to the P_z+_ state the magnetization in many domains rotates back towards [01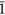] or [0
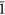
1] directions as indicated by both positive and negative XMCD asymmetry above 2 kV/cm (Fig. 5(b)). The magnetization in the entire field of view does not rotate at the same field, but forms stripes of magnetization oriented along the [01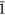] direction. Thus, the PMN-PT FE switching field has a large spatial variation, and careful spatially resolved analysis is necessary to distinguish partially switched and fully switched FE configurations.

### Electric-field driven changes in free-energy density

**Figure 6:**
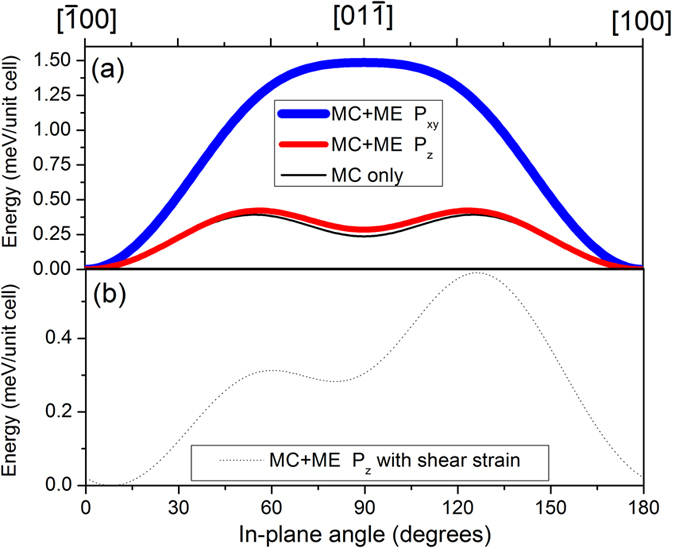
(**a**) Change in free energy density as a function of in-plane angle and dilational strain values 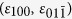 determined from X-ray diffraction for the P_xy_ and P_z_ states. While the P_xy_ state has a uniaxial magnetic easy axis parallel to [100], the P_z_ state has stable magnetization along both [100] and [01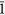] directions, resulting in a weakly four-fold in-plane magnetic easy symmetry. (**b**) A small shear strain of 3 × 10^−4^ induces a rotation of the stable magnetization points by 8° and −10° for the [100] and [01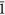] stable magnetization directions, respectively.

We calculate the contributions to the free energy density^29^ stemming from magnetocrystalline (MC) and magnetoelastic (ME) anisotropy terms to determine to what degree a change in strain can induce magnetization rotation in the LSMO film. Magnetostriction constants for manganites in both bulk and thin film form have been found to range from 10^−5^ to 10^−4 ^27,30,31, leading to a significant magnetoelastic anisotropy in the limit of weak magnetocrystalline anisotropy. Figure 6(a) plots the free energy density as a function of in-plane magnetization angle and experimentally determined dilational thin film strain (see Supplementary Figure S4 for the variation of magnetic easy axis with anisotropic strain). In the P_xy_ state (thick blue curve), the ME energy term is large compared to the MC term, there are two energy minima at the [100] and 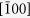 directions, and a large energy barrier along the [01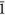] direction necessitating a large reversal magnetic field for 180° magnetization reversal. On the other hand, the P_z_ state (red curve) has only a slight contribution from ME energy and is dominated by the MC energy term contribution (thin black curve). A local minimum at the [01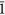] direction suggests that magnetization reversal can occur from [100] to 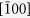 through an intermediate 90° rotation with magnetization along a metastable [01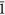] direction. Furthermore, the reduction of in-plane strain along the [01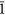] direction on changing from a P_xy_ to P_z_ state (see Supplementary Table S1) reduces the free energy density for LSMO magnetization along the [01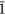] direction by more than 70% (from 1.5 meV/unit cell to 0.28 meV/unit cell). This leads to possible stable magnetization along both 〈01
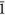
〉 and 〈100〉 type directions for the P_z_ poled state.

Additionally, shear strains generated due to a change in in-plane unit cell symmetry imposed by the substrate can shift the energy minimum and thus the easy in-plane magnetization direction (Fig. 6(b)). For manganite films grown on (011)-oriented SrTiO_3_ substrates^32^, an in-plane shear strain of up to 0.01 was measured due to templating of the rhombohedral LSMO onto a cubic unit cell. For our LSMO/PMN-PT sample we expect a smaller shear strain due to the non-cubic PMN-PT crystal symmetry as well as the large epitaxial mismatch (Supplementary Table S2), but it is difficult to determine the shear strain from X-ray diffraction measurements due to averaging over an ensemble of many domains. Figure 6(b) shows a magnetization rotation of up to 10 degrees for a modest shear strain of 3 × 10^−4^, smaller than the angular distortion of the PMN-PT surface unit cell generated upon FE axis rotation of approximately 1 × 10^−3^. Thus, dilational or shear strain changes in the LSMO film can induce large magnetization angle rotations as well as affect the barrier height for 180° magnetization reversal.

## Discussion

From both film-averaged and spatially resolved measurement techniques, we directly link the change in LSMO unit cell dimensions induced by poling of the PMN-PT substrate from P_xy_ to P_z_ states or vice versa to the changes in magnetic anisotropy symmetry and in-plane magnetic easy axis direction rotation as imaged on the length scale of a single magnetic domain through PEEM. The locally observed domain evolution measured in PEEM images taken at remanence coincides with the domain averaged XMCD hysteresis loops measured in an applied magnetic field. It is the lowered energy barrier for domain nucleation as well as the lowered energy barrier needed to rotate moments to 〈01
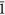
〉 directions that leads to the significantly different magnetic hysteresis behavior between the P_xy_ and P_z_ states. Small changes in strain state between an applied field of −6.5 kV/cm and 1 kV/cm due to the converse piezoelectric effect lead to propagation of domains walls on the scale of 1 μm (see Supplementary Movie S1), showing that in addition to the anisotropy tuning obtained from changing strain symmetry, ferromagnetic domain wall manipulation is possible at electric fields smaller than 1 kV/cm.

We have shown that this perovskite artificial multiferroic system can show significant non-volatile room temperature modulation of ferromagnetic domain walls, magnetic anisotropy and resistivity through the careful selection of ferroelectric and ferromagnetic crystal orientation. In particular, domain-resolved imaging of the magnetization shows a marked change in magnetic switching behavior due to the change in imprinted strain state between ferroelectric substrate and ferromagnetic film. Calculation of the change in free energy density due to this imprinted strain state confirms the significant lowering of energy barrier for magnetization reversal, as well as showing that a weak magnetocrystalline anisotropy combined with anisotropic changes in strain are responsible for the change between two-fold and four-fold magnetic easy axes as a function of electric field. This added breadth of functionality when compared to artificial multiferroic systems with polycrystalline metal ferromagnetic elements opens up the possibility of multifunctional low-power room temperature nanostructures and domain-wall devices that take advantage of both of the magnetic anisotropy, domain nucleation barrier lowering, and resistive tuning degrees of freedom and whose functional states may be written and reset with modest electric field pulses.

## Methods

An LSMO layer of 17 nm thickness was grown on a polished (011)-oriented PMN-PT substrate via pulsed laser deposition. The substrate was held at a temperature of 660 °C in a 300 mTorr oxygen ambient, and laser fluence and repetition rate were 1.2 J/cm^2^ and 1 Hz, respectively. The sample was cooled in a 300 Torr oxygen ambient at 8 °C/min. For poling experiments, the LSMO film served as a top contact while a 40 nm gold counter-electrode was sputtered on the back side of the PMN-PT substrate to serve as a bottom contact.

High resolution X-ray diffraction characterization was performed at room temperature using a Bruker D8 Discover system equipped with a monochromatized Cu K_α1_ source.

Resistivity measurements were performed in a customized Lakeshore TTPX probe station in the van der Pauw geometry with a Kepco bipolar power amplifier as the voltage source for poling the PMN-PT substrate. Note that due to the different electrode-FE interfaces (i.e. the LSMO/PMN-PT compared to the PMN-PT/Au interface), an asymmetry in the resistivity hysteresis loops is observed due to non-symmetric FE reversal processes.

Temperature- and magnetic field-dependent magnetization measurements were performed in a Quantum Design MPMS XL magnetometer with the magnetic field along either the in-plane [100] or [01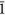] crystallographic directions of the sample. Measurements at 298 K verify the trend with XMCD hysteresis loops, with *S*_100_ = 0.59 and 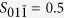 for the out-of-plane poled P_z_ state 10 weeks after a pulsed field of 6 kV/cm, showing clear retention of the magnetic anisotropy for a significant duration after the electric field pulse.

The change in free energy from magnetocrystalline and magnetoelastic anisotropy terms as a function of in-plane angle and strain^33^,^34^,^35^ were performed by using average values for manganite compliance tensor terms at 298 K (c_11_ = 200 GPa, c_12_ = 110 GPa, c_44_ = 45 GPa)^24^,^36^ and magnetocrystalline anisotropy terms determined from torque magnetometry at room temperature (K_1_ = 2.6 kJ/m^3^ and K_2_ = 5.7 kJ/m^3^)^27^. The in-plane angle locations of stable energy minima were found from the second derivative of the free energy (see Supplementary Information).

PEEM imaging at the Mn L_3_ edge was performed at the Surfaces/Interfaces: Microscopy beamline at the Swiss Light Source^37^, with magnetic domain structure obtained by taking the XMCD asymmetry of images taken with right and left circularly polarized X-rays (*I*_*RCP*_ − *I*_*LCP*_)/(*I*_*RCP*_ + *I*_*LCP*_). X-ray absorption spectroscopy and hysteresis measurements were performed at beamlines 6.3.1 and 4.0.2 of the Advanced Light Source^38^,^39^. The LSMO sample surface was kept at ‘ground’ with respect to standard measurements (−20 kV for PEEM measurements, −50 V for X-ray absorption measurements), with a custom power supply^40^ or Keithley 6487 source applying voltage to the back side gold contact, respectively. The sample was measured in electron yield mode for all measurements as a function of azimuthal angle *ϕ*, with an X-ray grazing incidence angle *θ* of 30° from the surface for spectroscopy and hysteresis measurements and 16° for PEEM measurements. While imaging, the sample must be measured in magnetic remanence due to the deflection of secondary electrons by any large external magnetic fields such as from the electromagnet in the PEEM sample holder.

## Additional Information

**How to cite this article**: Chopdekar, R. V. *et al.* Giant reversible anisotropy changes at room temperature in a (La,Sr)MnO_3_/Pb(Mg,Nb,Ti)O_3_ magneto-electric heterostructure. *Sci. Rep.* **6**, 27501; doi: 10.1038/srep27501 (2016).

## Supplementary Material

Supplementary Information

Supplementary Movie 1
