# Supplementary material for: Giant reversible anisotropy changes at room temperature in a (La,Sr)MnO3/Pb(Mg,Nb,Ti)O3 magneto-electric heterostructure
Source: Sci Rep. 2016 Jun 8;6:27501. doi: 10.1038/srep27501 (PMC4897745; doi:10.1038/srep27501)
Supplement: Supplementary Information [file srep27501-s1.pdf]

Supplementary Information for “Giant reversible anisotropy changes at room temperature in a $(La,Sr)MnO_3/Pb(Mg,Nb,Ti)O_3$ magneto-electric heterostructure”

Rajesh Vilas Chopdekar[1], Michele Buzzi[2], Catherine Jenkins[3], Elke Arenholz[3], Frithjof Nolting[2], and Yayoi Takamura[1]

[1] Department of Materials Science and Engineering, Univ. of California, Davis, Davis, CA 95616, USA

[2] Swiss Light Source, Paul Scherrer Institute, CH-5232 Villigen PSI, Switzerland

[3] Advanced Light Source, Lawrence Berkeley National Laboratory, Berkeley, CA 95616, USA

A. X-ray diffraction characterization and dynamical tuning of transition temperatures

The poled PMN-PT substrate in this work can be indexed at room temperature to a monoclinic unit cell with a small deviation from orthorhombic (β~89.86° for a similar composition).[1] For a ferroelectric polarization along a [111] direction, there are {011} planes that have a partial projection of the indicated [111] direction and {011} planes that fully contain the [111] direction. For an applied electric field along the [011] direction, we can examine the possible out-of-plane and in-plane lattice dimensions as a function of poling state and calculate how much change in epitaxial mismatch can be generated by rotation of the ferroelectric (FE) axis from partially out of plane to wholly in the (011) plane.

X-ray diffraction characterization of the LSMO/PMN-PT sample using a lab diffractometer (Bruker D8 Discover) was performed to evaluate the change in film and substrate lattice parameters as a function of applied electric field. Lattice parameters for the LSMO film determined from reciprocal space maps, and electric-field induced changes in unit cell dimensions are presented in Table S1. Differences between the changes in lattice parameter from X-ray diffraction results as compared to the macroscopic strain gauge results from Ref. 2 may be due to partial loss of strain transfer through strain gauge adhesive or similar effects.

Crystallographic Direction	Strain gauge on PMN-PT	PMN-PT substrate	LSMO film
In-plane [100]	*-150 ppm*	-400 ppm	-170 ppm
In-plane $[01\bar{1}]$	*1300 ppm*	2300 ppm	2200 ppm
Out-of-plane [011]		-1100 ppm	-800 ppm

Table S1 – Spatially averaged change in dimension along orthogonal directions of a PMN-PT substrate measured between the P_{xy} and P_z configurations in zero electric field as measured by a strain gauge (from Wu *et al.*[2]) compared to the change in the same directions determined from X-ray diffraction reciprocal space maps for the LSMO/PMN-PT sample.

Figure S1(a) illustrates the change in out of plane $\{220\}$ peak intensity for the PMN-PT substrate, with the peaks indicated by vertical lines. Here we plot ω-2θ line scans as a function of substrate poling state as the ω-2θ scans clearly show the electric-field induced changes in relative peak intensity and thus changes to the FE domain populations.

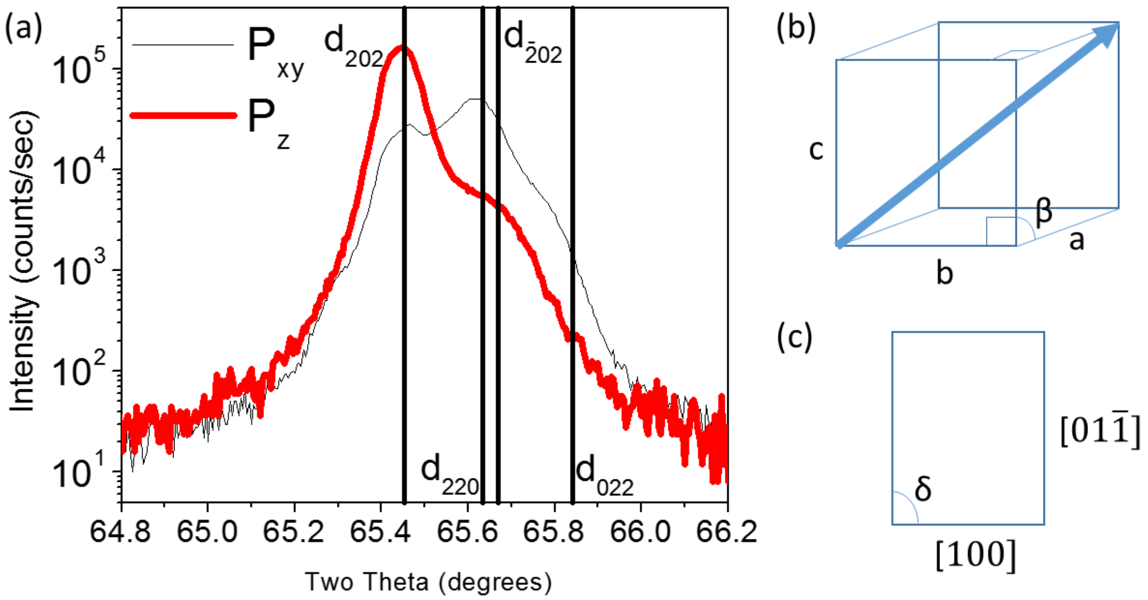

Figure S1 – (a) Out-of-plane X-ray diffraction ω-2θ scans near the $\{220\}$ peaks for a PMN-PT substrate as a function of poling state, with vertical lines corresponding to spacings as specified in Table S2. (b) Schematic of a monoclinic cell with a body diagonal indicated by bold arrow and (c) 011 projection of the monoclinic cell.

In Table S2, we tabulate the $\{220\}$ PMN-PT unit cell parameters found in reference 1, the corresponding 2θ angle, as well as epitaxial mismatch to a fully strained (011)-oriented LSMO. While one might expect all possible orientations present in a thermally randomized sample, we have determined from Figure S1 that the predominant in-plane orientations for the PMN-PT P_z and P_{xy} poling states after electric field cycling correspond to d_{202} and d_{220}, respectively (I_{202}/I_{total} = 95% for P_z, (I_{220} or $I_{\bar{2}02}$)/ I_{total} = 70% and I_{022}/I_{total} = 5% for P_{xy}). The monoclinic orientation in the P_z poling state is straightforward to understand – with a large electric field along the out of plane [011] direction, all FE domains will rotate to align their FE axes with the electric field, and the out of plane [011] length will be large compared to the in-plane $[01\bar{1}]$ direction. For the P_{xy} poling state, the FE axis rotates to lie in the (011) plane, so elongation along either the [100] or $[01\bar{1}]$ directions is possible.

Orientation	[100] length (Å)	$[01\bar{1}]$ length (Å)	[011] length (Å)	δ (deg)	2θ (deg)	Epitaxial mismatch along [100] (%)	Epitaxial mismatch along $[01\bar{1}]$ (%)
$\boldsymbol{d_{202}}$	**4.002**	**5.685**	**5.699**	**90**	**65.45**	**-3.09**	**-3.54**
$\boldsymbol{d_{022}}$	4.034	5.669	5.669	89.9	65.84	-3.86	-3.27
$\boldsymbol{d_{\bar{2}02}}$	**4.016**	**5.682**	**5.682**	**89.9**	**65.67**	**-3.43**	**-3.49**
$\boldsymbol{d_{220}}$	**4.002**	**5.699**	**5.685**	**90**	**65.63**	**-3.09**	**-3.78**

Table S2 –PMN-PT monoclinic cell data from Ref 1 with the predominant orientations in Figure S1(a) bolded, tabulating in-plane and out-of-plane dimensions as well as in-plane angle, δ. The corresponding diffraction angle from the out-of-plane spacing is also indicated. For comparison, the epitaxial mismatch between (011)-oriented LSMO and PMN-PT unit cells along the orthogonal in-plane directions is also tabulated.

The last two columns of Table S2 show the epitaxial misfit strain between a pseudocubic LSMO unit cell and monoclinic PMN-PT unit cell for each of the possible in-plane {011} planes, and the most significant change between d_{202} and d_{220} is along the $[01\bar{1}]$ direction, whereas a change between d_{202} and $d_{\bar{2}02}$ is along the $[100]$ direction. To first order, we expect an anisotropic strain change in the LSMO unit cell on any single PMN-PT FE domain transitioning from P_z to P_{xy}, with a large change in either the in-plane $[01\bar{1}]$ or $[100]$ direction of more than 2400 ppm and little change along the orthogonal in-plane direction. The experimentally derived changes in lattice parameter for the LSMO film presented in Table S2 suggest that the dominant switching route between P_z and P_{xy} poling states is from d_{202} to d_{220} due to the large change along the $[01\bar{1}]$ direction and negligible change along the $[100]$ direction.

However, this is a simplification of the mismatch between the rhombohedral LSMO unit cell and the PMN-PT in-plane dimensions. For instance, PMN-PT compositions near the morphotropic phase boundary undergo electric-field induced changes in symmetry (e.g. from rhombohedral to orthorhombic), as well as phase transitions due to stress or temperature near ambient conditions.[3,4] Furthermore, the rhombohedral LSMO unit cell forms a microtwin structure when templating on a cubic (110)-oriented $SrTiO_3$ surface, [5] but a change in in-plane shear strain from the as-grown state on PMN-PT can be generated due to the change in in-plane angle δ (see Table S1) between the inequivalent [100] and the $[01\bar{1}]$ directions. Thus, for the (011) LSMO film, we can expect both elongation type strain along the direction as well as a contribution from shear strain as a function of PMN-PT poling state.

B. PEEM vector magnetometry mapping of the P_z state

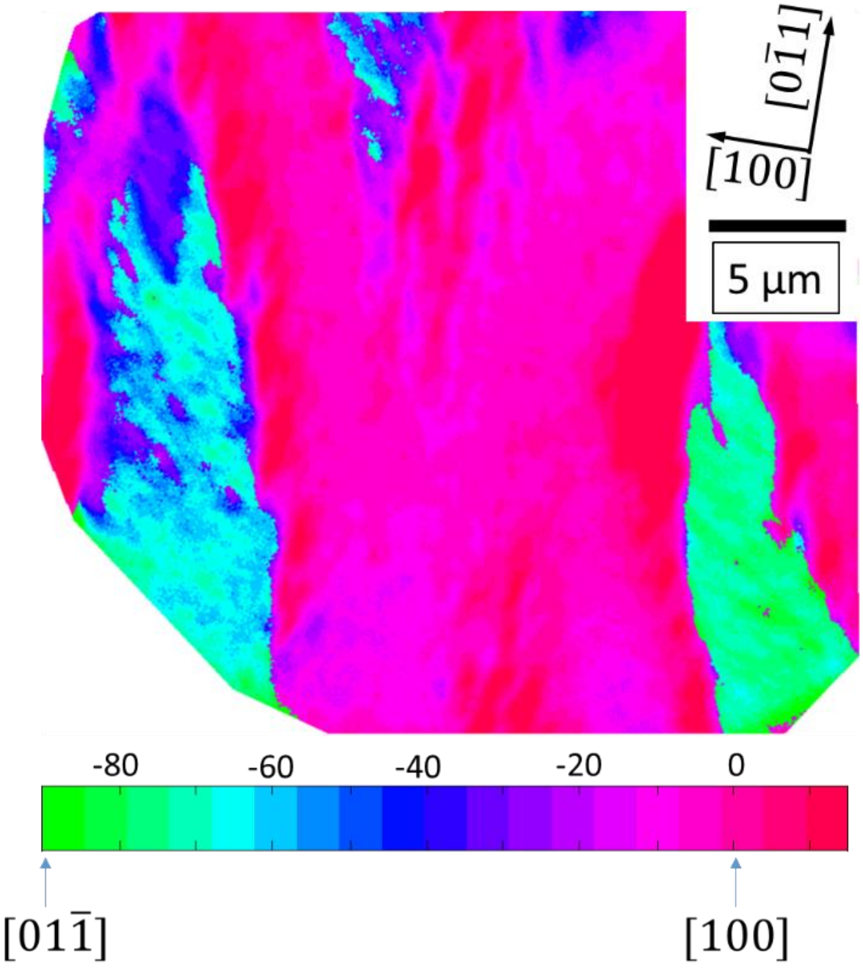

Figure S2 – In-plane magnetization direction for the LSMO thin film with the PMN-PT substrate poled in the P_z configuration. Domains show magnetization both along [100] and $[01\bar{1}]$ directions.

A series of PEEM-XMCD images as a function of in-plane azimuthal angle allows for the determination of both the magnitude and direction of the local sample magnetization. Figure S2 shows the in-plane magnetization direction for a sample poled in the P_z state after thermal demagnetization to 340 K and cooling to room temperature in zero magnetic field. PEEM-XMCD images were continuously acquired while heating and cooling to ensure the sample was heated at least 10 K above the point that all magnetic contrast was lost. Magnetic domains align both along the [100] and $[01\bar{1}]$ directions to first order, but a more careful comparison of the color levels shows variation in neighboring domains of 10 degrees (e.g. pink vs red domains are mostly oriented along the [100] but are canted away from this direction by ±10°).

C. XMCD images as a function of magnetic field for P_{xy} poling state

In the same sample location as Figure 4, the sample was poled into the P_{xy} state from the P_{z-} state and XMCD asymmetry images were taken during a magnetic field pulse sequence from negative to positive saturation along [100]. The magnetization strongly aligns with the <100> directions in contrast to the P_z state, and the field of view reverses through nucleation of a 180° reversal followed by domain wall motion. A 1 x 1 µm region was integrated as a function of magnetic field pulse and plotted as red circles in Figure 4 of the main text.

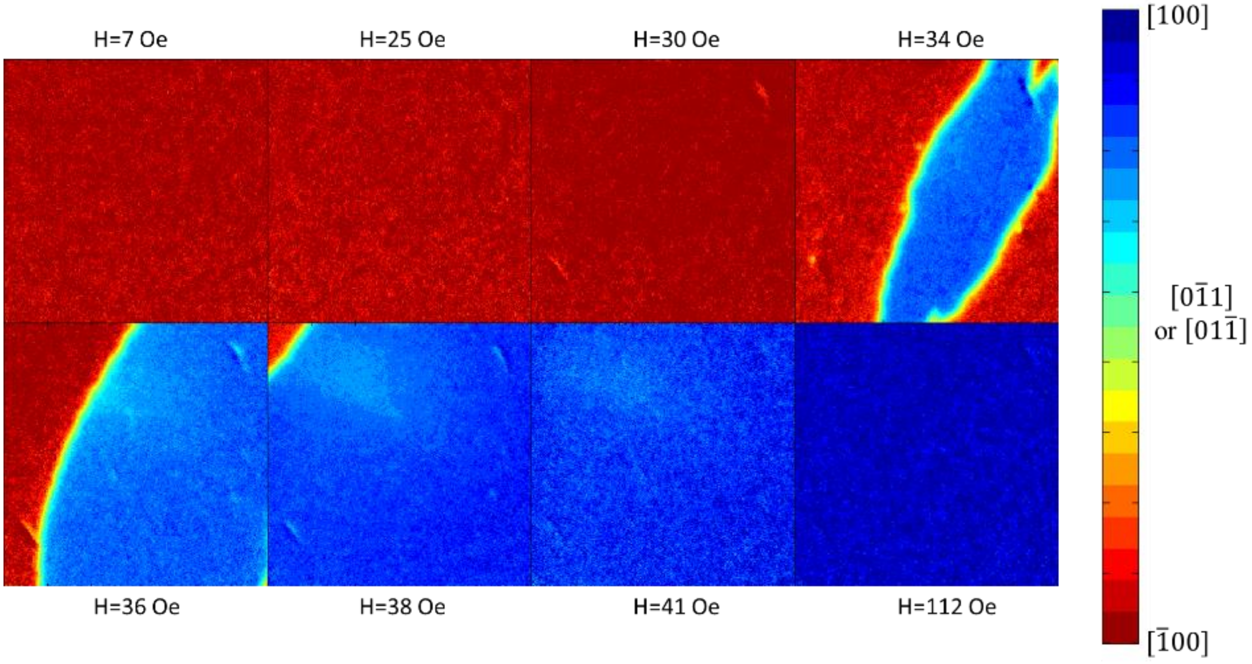

Figure S3 – Colorized XMCD images as a function of magnetic field pulse in the P_{xy} state showing 180° rotation of magnetization, in contrast to the non-180° rotation of magnetization for the P_z state shown in Figure 3.

D. Free energy calculation phase diagram

Figure S4 is a phase diagram of in-plane tensile strain for the (110)-oriented LSMO film with the experimentally determined normal strain values for the P_{xy} and P_z states indicated as points. For this map, we minimize the free energy f from magnetocrystalline and magnetoelastic terms to find stable magnetization angles ϕ in the sample plane:

$$f(\epsilon_{100}, \epsilon_{0\bar{1}1}, \phi) = K_1(\alpha_1^2\alpha_2^2 + \alpha_1^2\alpha_3^2 + \alpha_2^2\alpha_3^2) + K_2\alpha_1^2\alpha_2^2\alpha_3^2 + E_{ME}(\epsilon_{100}, \epsilon_{0\bar{1}1}, \lambda_s, \phi)$$

with α_i as direction cosines of the magnetization with respect to the orthogonal in-plane directions of the (011)-oriented film,[6] and the magnetoelastic energy term taken from Gao *et al* for a magnetostrictive film on a (011)-oriented substrate.[7] We first assume negligible in-plane shear strains, compliance tensor components as detailed in the methods section, and an isotropic magnetostriction constant of $\lambda_s = -1 * 10^{-5}$ at 300 K.[8-10]

Examination of anisotropic and bulk magnetostriction constants for LSMO single crystals show that anisotropic effects are negligible within 30 K of the Curie temperature, whereas bulk magnetostriction increases significantly in magnitude near the Curie temperature.[11] The boundaries are generated from the second derivative of the free energy density. The slopes of the phase boundaries are proportional to unity, thus a large anisotropic strain in either direction can induce a strongly uniaxial magnetic easy axis, but nearly isotropic strain allows for the magnetocrystalline anisotropy to dominate and the magnetic easy axis has a fourfold symmetry.

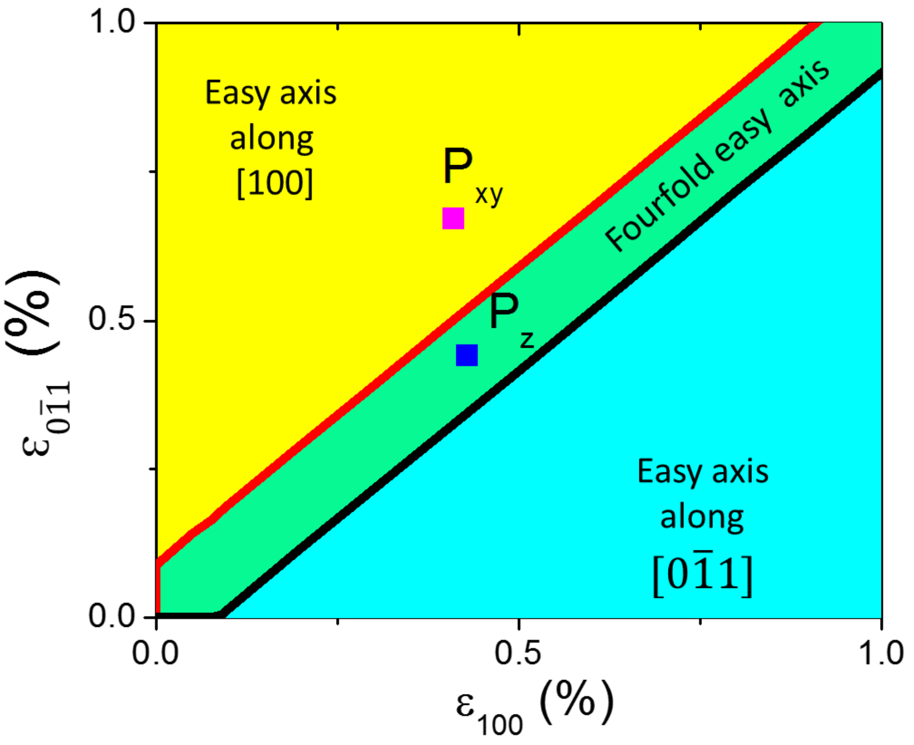

Figure S4 – Magnetic easy axis phase diagram as a function of in-plane strain along the two inequivalent crystallographic directions.

E. Supplementary Movie 1

Supplementary Movie 1 is a series of PEEM XMCD asymmetry images of a 30 micron field of view area used to generate the plot in Figure 5 (a). There are small changes in magnetic domain configuration as the electric field across the PMN-PT substrate is changed from -6.25 kV/cm to 1 kV/cm, but the largest rotation of magnetization occurs at approximately 1 kV/cm and 2.2 kV/cm as detailed in the main text – these changes correspond to the ferroelectric domain rotation from P_{z-} to P_{xy} and P_{xy} to P_{z+}, respectively. Piezoelectric-induced motion of the field of view or sample surface voltage fluctuations during image acquisition results in incomplete subtraction of topographical features when calculating XMCD asymmetry images, resulting in small black/white spots in some frames.

F. Surface roughness of LSMO/PMN-PT sample

The topography of the LSMO/PMN-PT sample was investigated after X-PEEM measurements through tapping mode atomic force microscopy (Veeco Multimode). A typical 3 micron x 3 micron region is shown in Figure S5, with surface roughness values below 1nm, similar to that found for PMN-PT substrate surfaces.[12]

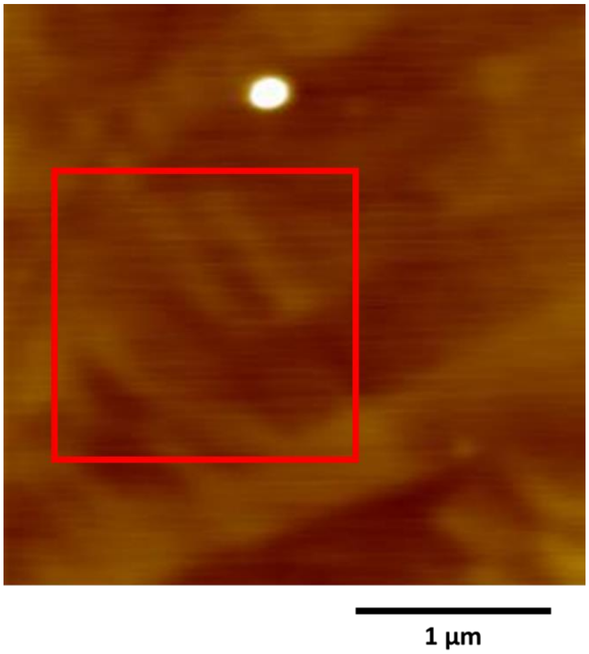

Figure S5 – Atomic force microscope image of the LSMO/PMN-PT sample, with the entire image having an RMS surface roughness of 0.65 nm, and region inside the red box having an RMS roughness of 0.45 nm.

References

1 Singh, A. K. & Pandey, D. Structure and the location of the morphotropic phase boundary region in (1- x)[Pb(Mg 1/3 Nb 2/3)O 3]- x PbTiO 3. *Journal of Physics: Condensed Matter* **13**, L931 (2001).

2 Wu, T. *et al.* Domain engineered switchable strain states in ferroelectric (011) [Pb(Mg1/3Nb2/3)O3](1−x)-[PbTiO3]x (PMN-PT, x≈0.32) single crystals. *Journal of Applied Physics* **109**, 124101 (2011).

3 McLaughlin, E. A., Liu, T. & Lynch, C. S. Relaxor ferroelectric PMN-32%PT crystals under stress and electric field loading: I-32 mode measurements. *Acta Materialia* **52**, 3849-3857 (2004).

4 Peräntie, J., Hagberg, J., Uusimäki, A., Tian, J. & Han, P. Characteristics of electric-field-induced polarization rotation in ⟨001⟩-poled Pb(Mg1/3Nb2/3)O3-PbTiO3 single crystals close to the morphotropic phase boundary. *Journal of Applied Physics* **112**, 034117 (2012).

5 Farag, N., Bobeth, M., Pompe, W. & Romanov, A. E. Modelling of structural domains and elastic strain calculation in rhombohedral La1−x Sr x MnO3 films on (110) SrTiO3. *Philosophical Magazine* **87**, 823-842 (2007).

6 Paes, V. Z. C. & Mosca, D. H. Effective elastic and magnetoelastic anisotropies for thin films with hexagonal and cubic crystal structures. *Journal of Magnetism and Magnetic Materials* **330**, 81-87 (2013).

7 Gao, Y., Hu, J., Shu, L. & Nan, C. W. Strain-mediated voltage control of magnetism in multiferroic Ni77Fe23/Pb(Mg1/3Nb2/3)0.7Ti0.3O3 heterostructure. *Applied Physics Letters* **104**, 142908 (2014).

8 Darling, T. W. *et al.* Measurement of the elastic tensor of a single crystal of La0.83Sr0.17MnO3 and its response to magnetic fields. *Physical Review B* **57**, 5093-5097 (1998).

9 Rajendran, V., Muthu Kumaran, S., Sivasubramanian, V., Jayakumar, T. & Raj, B. Anomalies in elastic moduli and ultrasonic attenuation near ferromagnetic transition temperature in La0.67Sr0.33MnO3 perovskite. *physica status solidi (a)* **195**, 350-358 (2003).

10 Suzuki, Y., Hwang, H. Y., Cheong, S.-W. & van Dover, R. B. The role of strain in magnetic anisotropy of manganite thin films. *Applied Physics Letters* **71**, 140-142 (1997).

11 Demin, R. V., Koroleva, L. I. & Balbashov, A. M. Anomalies of magnetostriction and thermal expansion in La0.7Sr0.3MnO3 perovskite. *Journal of Magnetism and Magnetic Materials* **177–181, Part 2**, 871-872 (1998).

12 Wu, T. *et al.* Electrical tuning of metastable dielectric constant of ferroelectric single crystals for low-power electronics. *Applied Physics Letters* **99**, 182903 (2011).
